# Surface Modification of Magnesium Oxysulfate Whisker Based on SiO_2_@silane Coupling Agent and SiO_2_@polydopamine Double-Layer Structure for Reinforcing HDPE

**DOI:** 10.3390/ma15093272

**Published:** 2022-05-02

**Authors:** Xiaochen Zhou, Yao Zhang, Guodong Jiang

**Affiliations:** College of Materials Science and Engineering, Nanjing Tech University, Nanjing 211816, China; zxc18796931434@163.com (X.Z.); zyrzy1314@163.com (Y.Z.)

**Keywords:** magnesium oxysulfate whisker (MOSw), polydopamine, γ-methacryloxypropyltrimethoxy (KH570), SiO_2_, high-density polyethylene (HDPE), mechanical properties, flame retardant

## Abstract

In this study, we fabricated high-performance polyethylene composites by constructing SiO_2_@silane coupling agent (γ-methylacryloxypropyl trimethoxysilane) and SiO_2_@polydopamine (PDA) double-layer structures on a magnesium oxysulfate whisker surface. In addition to realizing strong mechanical properties, the flame-retardant properties of the composites were effectively improved. Further increase in the initial crystallization temperature of the modified composites indicated that the dispersion of whisker in the matrix was improved. The drag effect of the modified whisker on the HDPE molecular chain was characterized by dynamic mechanical thermal analysis (DMTA) and the morphology of the impact-fractured surface was characterized by scanning electron microscopy (SEM); both confirmed the improved compatibility between the whisker and the matrix. The tensile strength of HDPE/MOSw@SiO_2_@KH570 and HDPE/MOSw@SiO_2_@PDA composites were 22.6% and 41.5% higher than that of the HDPE/MOSw composites, respectively. The impact strengths of the HDPE/MOSw@SiO_2_@KH570 and HDPE/MOSw@SiO_2_@PDA composites were 129% and 102% higher than that of the HDPE/MOSw composites, respectively. A stable carbon-silicate layer constructed by a SiO_2_@KH570 and SiO_2_@PDA double-layer structure delayed the combustion process. As a result, the limiting oxygen index (LOI) of HDPE/MOSw@SiO_2_@KH570 and HDPE/MOSw@SiO_2_@PDA composites increased from 22.5 to 22.9 and 23.5, respectively.

## 1. Introduction

Polyethylene (PE) [1,2] and polypropylene (PP) [3,4] are currently the most widely used thermoplastic materials. As their structures only contain carbon and hydrogen elements, they are easy to burn which creates safety risks. Therefore, it is necessary to make these materials flame-retardant [5,6] to broaden their fields of application.

As a new type of needle-fiber filler, magnesium oxysulfate whisker (MOSw) [7] has the characteristics of high strength, high modulus, and excellent dimensional stability. In addition, compared with common inorganic fillers, such as graphite and talc, MOSw has a lower decomposition temperature (the initial decomposition temperature is about 300 °C). One mole of magnesium oxysulfate whisker contains three moles of crystal water and five moles of water. The whiskers can quickly decompose at relatively low temperatures to generate a large amount of water vapor, which effectively dilutes the oxygen concentration around the substrate [8]. This process, due to the volatilization of adsorbed water, absorbs a lot of heat, effectively reducing the surrounding environment’s temperature, leading to a good flame-retardant effect. Therefore, in the development of polymer composite materials, MOSw has attracted popular attention as a substitute for glass fibers [9].

The general flame retardant modification method is to fill the matrix with a large amount of flame retardant, which often leads to a sharp decline in the mechanical properties of composites. Coordinating the relationship between the two to optimize both flame-retardant and mechanical performance has long been a major goal of plastics engineering. Filled polymers often need uniform and stable dispersion of the filler in the matrix and compatibility of the filler and the matrix [10,11,12,13,14]. MOSw has a smooth surface and is relatively polar due to the presence of hydroxyl groups and water of crystallization, so there is a problem of poor compatibility with most organic polymers. Therefore, surface treatment is needed to improve the compatibility [15,16,17] between the whisker and the resin.

At present, the surface treatment of filler is mainly divided into physical coating and chemical modification. The surface modification of MOSw is mainly achieved by silane coupling agents, titanate coupling agents, fatty acids, and other surfactants. Eui-Su Kim used 3-methacryloxy propyl-trimethoxy silane (MPS) for the surface treatment of MOSw [18]. The yield strength and elastic modulus of the modified composite were increased by 16.5% and 17.3% compared with those before modification. The PP limiting oxygen index (LOI) increased from 18% to 19.3%. Dang Li [19] studied the surface treatment of whiskers by various fatty acids and found that whiskers treated with lauric acid had the best dispersibility and hydrophobicity. When the whisker loading amount was 5wt%, the tensile strength of the PP composites treated with lauric acid increased by 8.5% compared with that of unmodified PP composites. In addition, the whiskers treated with dodecyl dihydrogen phosphate (DDP) had a better flame-retardant effect [20]. The LOI of PP increased from 18% to 26.1% when the whisker loading was 30 wt.%. Chenxi [21] used bis[3-(triethoxysily)propyl]tetrasulfide (Si69) for the surface treatment of MOSw. When the whisker loading amount was 4 wt.%, the tensile strength and elastic modulus of modified natural rubber (NR) composites were 34.4% and 20% higher than those before modification.

Research on MOSw-reinforced and flame-retardant polymers is mainly aimed at PP [22], ABS [23], and NR, with a few studies on PE. Therefore, this study focuses on the effect of basic magnesium sulfate whiskers on the enhancement and flame retardancy of HDPE.

In this study, sodium silicate (Na_2_SiO_3_) was used as the precursor of silicon dioxide to prepare the SiO_2_ layer on the surface of MOSw. To further improve the dispersion of whisker in the matrix and the compatibility between the whisker and the matrix, the surface treatment of γ-methacryloxypropyltrimethoxy silane (KH570) and polydopamine (PDA) was carried out based on SiO_2_ surface-coating to prepare MOSw@SiO_2_@KH570 and MOSw@SiO_2_@PDA. HDPE/MOSw, HDPE/MOSw@SiO_2_, HDPE/MOSw@SiO_2_@KH570, and HDPE/MOSw@SiO_2_@PDA composites were prepared by the melt blending method. The surface properties of MOSw before and after modification were compared by X-ray photoelectron spectroscopy (XPS) and X-ray diffraction (XRD). The effect of the surface treatment on the compatibility between the whisker and HDPE was appraised in terms of mechanical properties and by dynamic mechanical thermal analysis (DMTA). In addition, the influence of MOSw on the flame retardancy of HDPE before and after modification was studied by limiting oxygen index (LOI) and thermogravimetric analysis (TGA).

## 2. Materials and Methods

### 2.1. Materials

γ-Methacryloxypropyltrimethoxy silane (KH570) was obtained from the Shanghai Yuanye Biotechnology Co., Ltd., Shanghai, China. High-density polyethylene (HDPE) powder, 5000S, MI (melt index) = 0.9, was supplied from the Sinopec Yangzi Petrochemical Company, China. Magnesium oxysulfate whisker (MOSw) was supplied from the Sshanghai Fengzhu New Materials Technology Co., Ltd. 3-Hydroxytyramine hydrochloride (dopamine) and tris(hydroxymethyl) aminomethane (Tris) were obtained from the Meryer (Shanghai) Chemical Technology Co., Ltd., Shanghai, China. Sodium silicate (Na_2_SiO_3_) was obtained from the Shanghai Yien Chemical Technology Co., Ltd., Shanghai, China. Hydrochloric acid (HCl) was purchased from the Shanghai Lingfeng Chemical Reagent Co., LTD, Shanghai, China. Acetic acid was purchased from the Nanjing Haitai Scientific Equipment Co., LTD, Jiangsu, China.

### 2.2. Surface Modification of MOSw

**Figure 1:**
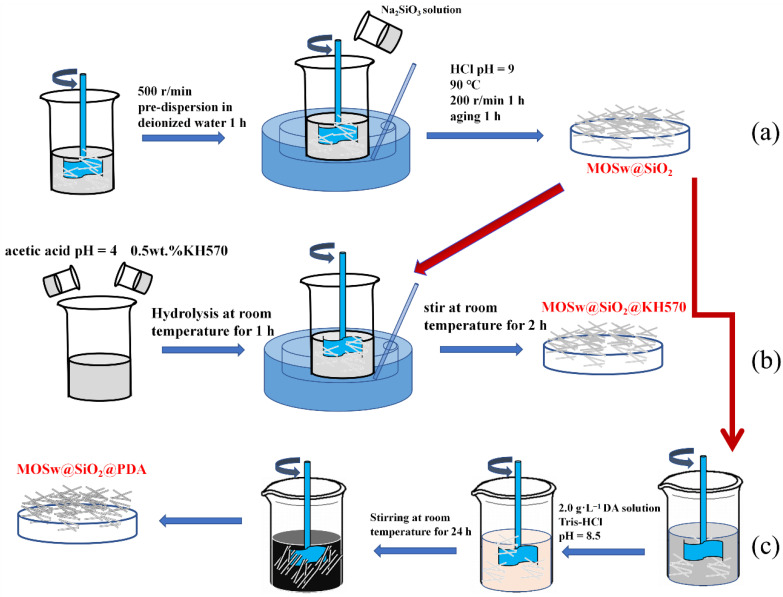
Preparation flowchart of (**a**) MOSw@SiO_2_ (**b**) MOSw@SiO_2_@KH570 (**c**) MOSw@SiO_2_@PDA.

First, 0.15 mol/L Na_2_SiO_3_ solution was prepared for reserve. Then, the whisker was put into water for pre-dispersion. A corresponding content of sodium silicate (Na_2_SiO_3_) with silicon dioxide content (1 wt.% MOSw) was added as standard. As shown in Figure 1a, by dropping Na_2_SiO_3_ solution and using HCl to adjust the solution pH to 9, stirring at 90 °C for 1 h at low speed, and then aging for 1 h, MOSw@SiO_2_ was prepared.

#### 2.2.1. The Preparation of MOSw@SiO_2_@KH570

First, the silane aqueous solution was prepared by hydrolysis of the silane coupling agent (KH570) in deionized water (DI). DI water was adjusted to pH 4 using acetic acid, and 0.5 wt.% of the silane coupling agent (KH570) was added and hydrolyzed for 1 h at room temperature. As shown in Figure 1b, the MOSw@SiO_2_ was mixed with silane aqueous solution for 2 h at room temperature for the salinization of MOSw.

#### 2.2.2. The Preparation of MOSw@SiO_2_@PDA

A dopamine aqueous solution (2 g /L) was configured, and the pH was set to 8.5 by adding Tris buffer solution. As shown in Figure 1c, the MOSw@SiO_2_ was added to the above solution and stirred at room temperature for 24 h.

### 2.3. Preparation of HDPE/MOSw Composites

Before mixing, all materials were dried at 60 °C for 8 h to eliminate moisture. The unmodified MOSw and modified MOSw were mixed with HDPE in an internal mixer (W50EHT-3zones, Brabender, Germany) at 180 °C and run at 60 r/min for 15 min. The whisker loading was fixed at 30 wt.%, and HDPE/MOSw, HDPE/MOSw@SiO_2_, HDPE/MOSw@SiO_2_@KH570, and HDPE/MOSw@SiO_2_@PDA composite materials were prepared. Then, the blends were preheated at 180 °C for 3 min, hot-pressed at 10 MPa for 10 min, and cold-pressed at 10 MPa for 3 min to prepare the plates. Finally, the plates were cut to prepare the test samples. All samples were stored in a dry environment at room temperature.

### 2.4. X-ray Photoelectron Spectroscopy (XPS) Analyses

Elemental composition analysis of the samples was carried out using X-ray photoelectron spectroscopy (XPS, Thermo Scientific K-Alpha, Suzhou, China). The binding energy (BE) scale was calibrated concerning the C1s line at 284.5 eV.

### 2.5. X-ray Diffraction (XRD) Analyses

MOSw, MOSw@SiO_2_, MOSw@SiO_2_@KH570, and MOSw@SiO_2_@PDA samples were measured at room temperature using XRD-6000 equipment, operating at a voltage of 40 kV and 15 mA. The applied radiation from the target Cu Kα was nickel filtered (λ = 0.154 nm). The range of scattering angles (2θ) was 5° to 65° with a speed of 10°/min.

### 2.6. Scanning Electron Microscopy (SEM) Analyses

The morphology of the composites was observed by scanning electron microscopy (SEM, JEOLJSM-650, Tokyo, Japan).

### 2.7. Dynamic Mechanical Thermal Analysis (DMTA)

Dynamic mechanical thermal analysis (DMTA) of HDPE composites was performed using a TA Instruments Q800, within a temperature range from 30 °C to 120 °C, at a heating rate of 3 °C /min.

### 2.8. Thermal Analyses

Thermogravimetric analysis (TGA) was used to determine the thermal resistance and degradation of the neat HDPE and HDPE composites. TGA data was operated in the dynamic mode from 50 to 650 °C at a heating rate of 10 °C/min in an air atmosphere.

The crystallization and melt behaviors were analyzed by a differential scanning calorimeter (DSC, Q200) under a nitrogen atmosphere. First, the samples were rapidly heated to 200 °C from 30 °C with a 10 °C /min heating rate and maintained for 5 min. This procedure aimed to eliminate any thermal history of the sample. Then, the heated samples were cooled down to 30 °C at a cooling rate of 10 °C/min. The crystallization curves were obtained through the above process. After that, the cooled samples were heated again to 200 °C at a heating rate of 10 °C/min, and then the melt curves were obtained. The crystallinity (X_c_) was calculated according to the following equation [24].

(1) $$X_{c}=\frac{\Delta H_{m}}{\Delta H_{m0}\left(1-\emptyset_{f}\right)}\times 100\%$$

where ∆*H_m_* is the melting enthalpy, ΔH_m0_ is the standard enthalpy of HDPE (277.1 J/g), and ∅*_f_* is the mass fraction of MOSw.

### 2.9. Mechanical Properties

ASTM D882-2018 was adopted as the tensile test standard for composite materials. All samples with sizes of 100 mm × 10 mm × 0.3 mm were kept at a constant temperature of 25 °C for 24 h and then tested. The tensile properties of the material were measured at a rate of 2 mm/min using a CMT 5254 electronic universal testing machine at 25 °C. At least 5 samples were tested for each group; the final results were obtained by averaging.

Notched impact strength tests were conducted on a radial-boom impact tester (UJ-4, Chengde Testing Machine Manufacturing Co., Ltd., China) according to ISO 180. Before testing, a V-notched to a depth of 2 mm was machined on each specimen. In addition, all samples with sizes of 80 mm × 10 mm × 4 mm were kept at a constant temperature of 25 °C for 24 h and then tested. At least 5 samples were tested for each group; the final results were obtained by averaging.

### 2.10. Limit Oxygen Index (LOI) Analyses

Limiting oxygen index refers to the minimum oxygen concentration that can sustain combustion of the tested sample in an N_2_-O_2_ mixture under specified experimental conditions, expressed in terms of the percentage of oxygen by volume. According to the GB/T 2406 method, the limit oxygen index of the sample was measured by the HC-2 oxygen index tester. All samples with sizes of 80 mm × 10 mm × 4 mm were kept at a constant temperature of 25 °C for 24 h and then tested. At least 5 samples were tested for each group.

### 2.11. Contact Angle Analyses

The static contact angles of MOSw@SiO_2_@KH570 and MOSw@SiO_2_@PDA were measured at room temperature using a static contact angle tester (DSA 100).

## 3. Results

### 3.1. XPS Analysis

**Figure 2:**
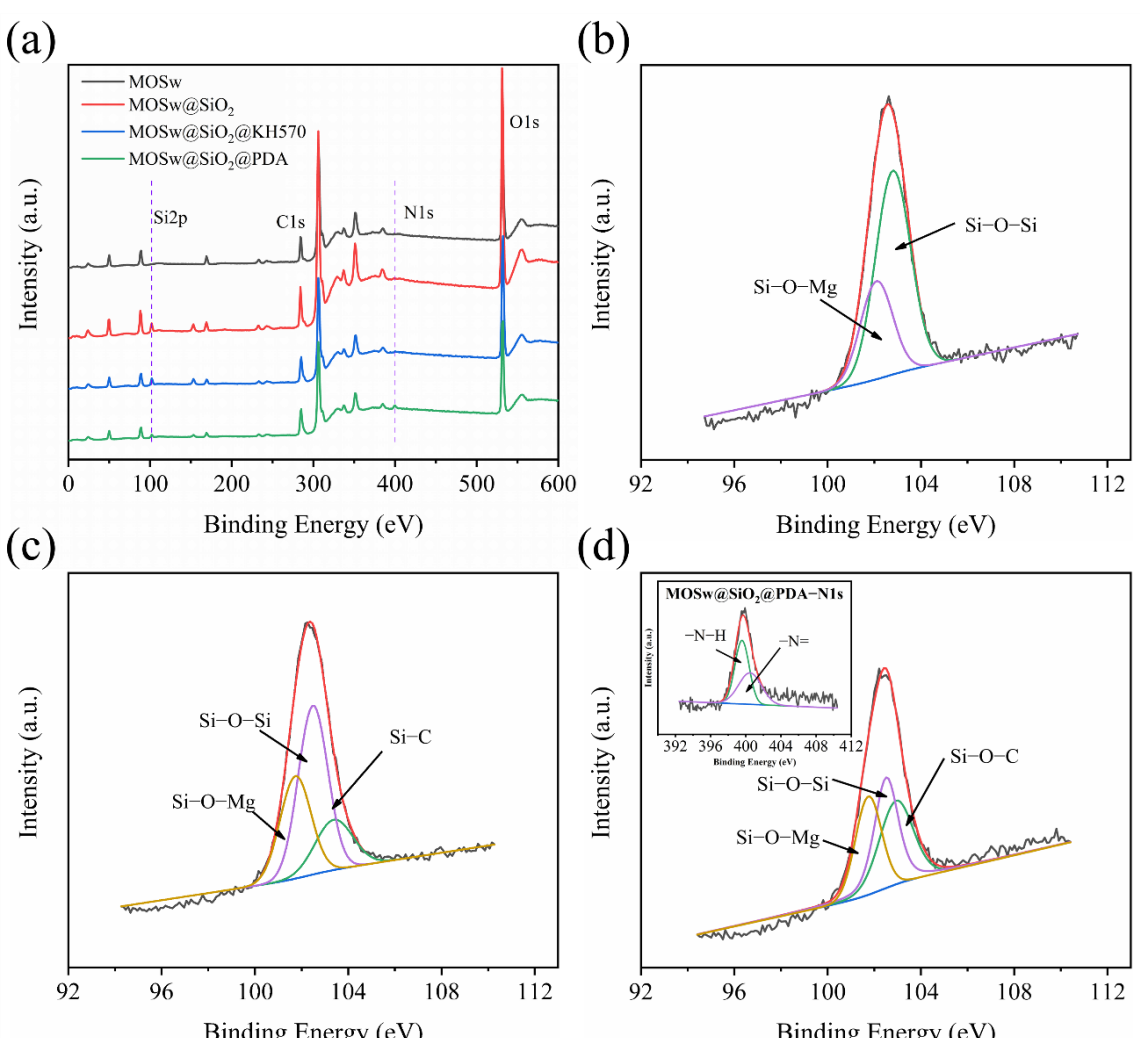
XPS spectra, Si2p for (**a**) Unmodified MOSw, (**b**) MOSw@SiO_2_, (**c**) MOSw@SiO_2_@KH570, (**d**) MOSw@SiO_2_@PDA.

Figure 2a shows the full spectrum of XPS before and after whisker modification. Table 1 shows the changes of elemental content on the whisker surface before and after modification. The presence of element C on the surface of MOSw and MOSw@SiO_2_ may have been due to air pollution caused by improper sample storage, resulting in the adsorption of carbon dioxide on the surface of MOSw [25]. From Figure 2a, it can be seen that the Si element was added to the whisker surface after it was coated with silica. Then the secondary treatment of KH570 caused the content of C and Si to increase again. The secondary treatment of PDA added the N element, and the increase in the C element content was attributed to the presence of a large number of benzene rings in polydopamine. The above phenomena preliminarily suggested that the three modification methods were successful. Figure 2b shows the XPS spectra of Si 2p on the surface of MOSw@SiO_2_. The peaks at 102.8 eV and 102.1 eV correspond to the binding energies of Si-O-Si and Si-O-Mg, respectively. The chemical bond energy of Si-O-Mg was mainly formed by the combination of OH groups on the whisker surface with Si(OH)_4_, and Si(OH)_4_ was mainly obtained by the hydrolysis of sodium silicate and the reaction with HCl. The chemical bond energy of Si-O-Si was formed by the condensation between Si(OH)_4_ and Si(OH)_4_. Figure 2c shows the XPS spectra of Si 2p on the surface of MOSw@SiO_2_@KH570. The peaks at 103.4 eV, 102.5 eV, and 101.7 eV correspond to the binding energies of Si-C, Si-O-Si, and Si-O-Mg [26], respectively. In addition to the condensation of Si(OH)_4_, the formation of chemical bond energy of Si-O-Si also included the condensation reaction between Si-OH after hydrolysis of KH570 and Si-OH of silicon dioxide. In addition, the formation of Si-C bond energy was mainly attributed to the Si-C bond existing in KH570 itself. Figure 2d shows the XPS spectra of Si 2p and N 1s on the MOSw@SiO_2_@PDA surface. The peaks at 102.9 eV, 102.5 eV, 101.8 eV, 399.6 eV, and 400.5 eV correspond to the binding energies of Si-O-C, Si-O-Si, Si-O-Mg, -N-H, and −N=, respectively. The formation of Si-O-C chemical bond energy was mainly through the combination of Si-OH on the surface of silicon dioxide and the OH group on the surface of polydopamine. The -N-H groups were due to the presence of dopamine, whereas the −N= groups were formed by the indole groups in the process of dopamine self-polymerization [24,27]. The above results are sufficient to demonstrate that the three modification methods were successful.

### 3.2. XRD Analysis

**Figure 3:**
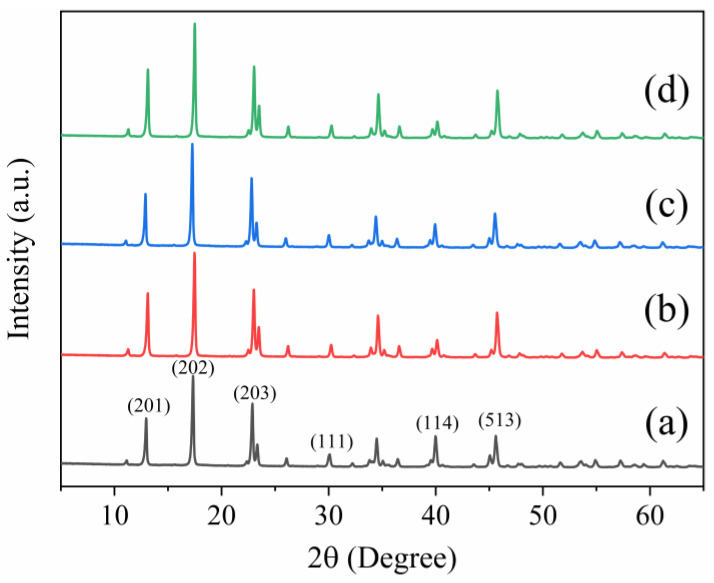
X-ray diffraction (XRD) curves, of (**a**) Unmodified MOSw, (**b**) MOSw@SiO_2_, (**c**) MOSw@SiO_2_@KH570, (**d**) MOSw@SiO_2_@PDA.

Figure 3 shows the XRD patterns of Unmodified-MOSw, MOSw@SiO_2_, MOSw@SiO_2_@KH570, and MOSw@SiO_2_@PDA. These four curves present the same diffraction peaks at 2θ around 13°, 17°, 23°, 30°, 34°, 40°, and 46°, assigned to (201), (202), (203), (111), (601), (114) and (513) [28] crystal faces, respectively. According to the peaks retrieved from PDF# 07-0415, the samples were orthorhombic MgSO_4_**·**5Mg(OH)_2_**·**3H_2_O. The results of the XRD characterization indicate that the crystal structure of MOSw treated by SiO_2_, SiO_2_@KH570, and SiO_2_@PDA, respectively, was not changed. At the same time, MOSw retained a high-quality single-crystal structure and maintained a high degree of crystallinity [29].

### 3.3. Mechanical Properties

**Figure 4:**
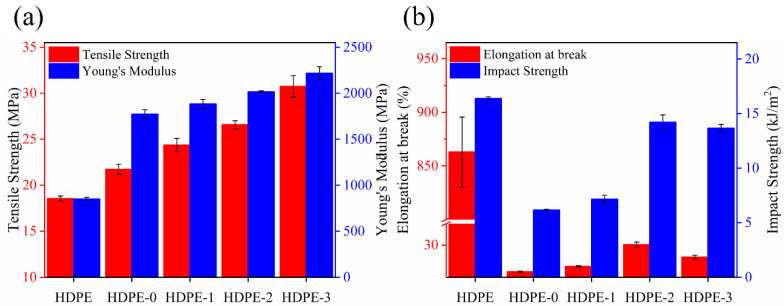
Schematic diagram of mechanical properties of HDPE, HDPE-0 (HDPE/MOSw), HDPE-1 (HDPE/MOSw@SiO_2_), HDPE-2 (HDPE/MOSw@SiO_2_@KH570), and HDPE-3 (HDPE/MOSw@SiO_2_@PDA) composites. (**a**) Tensile strength and Young’s modulus. (**b**) Elongation at break and impact strength.

The results of the tensile tests for the neat HDPE, HDPE/MOSw, HDPE/MOSw@SiO_2_, HDPE/MOSw@SiO_2_@KH570, and HDPE/MOSw@SiO_2_@PDA composites are listed in Figure 4. Compared to the neat HDPE polymer, the tensile strength of the HDPE/MOSw composites increased slightly, from 18.6 MPa to 21.7 MPa by 16.7% (Figure 4a). These results can be credited to the high strength and high modulus of MOSw as the filler having a higher stiffness than the matrix normally enhances the mechanical properties of composites [30].

Coating the whisker with SiO_2_ produced a positive effect on the tensile properties by its improvement of the interfacial bonding between MOSw and HDPE. Compared to the neat HDPE polymer, the tensile strength of the HDPE/MOSw@SiO_2_ composites increased slightly, increasing from 21.7 MPa to 24.4 MPa by 12.4%. Meanwhile, the Young’s modulus of the HDPE/MOSw@SiO_2_ composites was 6.1% higher than that of the HDPE/MOSw composites. The introduction of KH570 and PDA further improved the interfacial bonding force between the whisker and the matrix, so that the stress could be transferred from the matrix to the whisker, thus improving the tensile strength of the HDPE composites. The tensile strength of the HDPE/MOSw@SiO_2_@KH570 and HDPE/MOSw@SiO_2_@PDA composites was 22.6% and 41.5% higher than that of the HDPE/MOSw composites, respectively. Meanwhile, the Young’s modulus of the HDPE/MOSw@SiO_2_@KH570 and HDPE/MOSw@SiO_2_@PDA composites was 13.6% and 24.9% higher than that of the HDPE/MOSw composites, respectively.

Compared with HDPE/MOSw@SiO_2_@KH570, the tensile properties of the HDPE/MOSw@SiO_2_@PDA composites were more obviously improved, which was mainly attributed to the advantages of PDA secondary treatment in improving the interface binding force between the whisker and the matrix. DMTA analysis showed that the composites after PDA secondary treatment showed the highest energy storage modulus and loss modulus; that is, the interface binding force between the whisker and the matrix was the highest, which was more conducive to the transfer of stress, thus significantly improving the strengthening effect of the whisker on the matrix.

Just like general inorganic powder-filled polymers, the elongation at the break of the composite obviously decreased with the addition of MOSw. As the coating of SiO_2_ on the whisker surface improved the whisker agglomeration in the matrix, the elongation at the break of the composite increased accordingly. The secondary treatment of KH570 and PDA further improved the dispersibility of the filler in the matrix. In addition, the interfacial bonding force between the whisker and the matrix was greatly improved, which solved the problem of the whisker easily debonding with the matrix and causing the failure of the composite‘s properties. Therefore, the elongation at break of the composites after secondary modification increased by 200% (HDPE/MOSw@SiO_2_@KH570) and 87% (HDPE/MOSw@SiO_2_@PDA), respectively, compared with that before modification.

The influence of MOSw, before and after modification, on the impact properties of the polyethylene composites is shown in Figure 4b. It can be seen that the impact strength of the composites decreased dramatically with the addition of MOSw. The main reason was that, in the HDPE/MOSw composites, the agglomerated whisker acted as a stress concentration point, which increased the possibility of crack initiation. The cracks propagated rapidly around the poor interface regions, thus damaging the material properties with less energy. In addition, due to the weak interfacial bonding force between the whisker and the matrix, debonding occurred between the whisker and the matrix. Therefore, less energy was consumed in the impact process to destroy the material properties. The impact strength of the modified composite was obviously improved.

The impact strength of the HDPE/MOSw@SiO_2_, HDPE/MOSw@SiO_2_@KH570, and HDPE/MOSw@SiO_2_@PDA composites performed 16%, 129%, and 102% higher than that of the HDPE/MOSw composites, respectively. This was mainly due to the good dispersion of whisker in the matrix and the strong interfacial adhesion between the whisker and the matrix. Due to the addition of modifiers, the whisker agglomeration phenomenon was improved and the interface interaction between the whisker and the matrix was enhanced, which caused the initiation of cracks to consume more energy. In addition, the stronger interfacial bonding force caused the whisker to consume more energy in the process of pulling out. Therefore, the impact strength of the composite materials could be improved.

### 3.4. SEM Analysis

**Figure 5:**
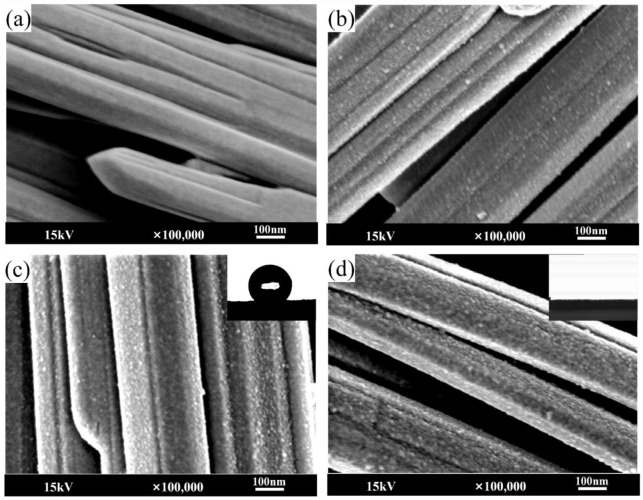
Images and SEM micrographs of (**a**) Unmodified MOSw, (**b**) MOSw@SiO_2_, (**c**) MOSw@SiO_2_@KH570, (**d**) MOSw@SiO_2_@PDA.

The surface morphology of MOSw before and after treatment was observed by SEM. It can be seen from Figure 5a that the unmodified MOSw displayed a smooth surface. A slightly coarse silica layer was deposited on the surface of the whisker by hydrolysis condensation of sodium silicate, as shown in Figure 5b. The modification mechanism was such that sodium silicate reacted with water to generate NaSiO_2_(OH)_2_, and Si(OH)_4_ was generated by neutralization of NaSiO_2_(OH)_2_ with hydrochloric acid. The formed Si(OH)_4_ was combined with the OH groups on the surface of MOSw and the SiO_2_ layer was formed on the surface of MOSw through a repeated condensation reaction [31]. Figure 5c–d show the whisker surface after secondary treatment of KH570 and PDA respectively, showing a rough surface coating layer. In addition, after secondary treatment with KH570, the whisker showed good hydrophobicity, and the contact angle reached 140.7°. This was mainly due to the substitution of Si-OH on the whisker surface by the lower surface energy groups Si-CH_3_ [32,33]. However, the secondary treatment of PDA introduced a large number of hydrophilic groups, so the whisker surface still showed high hydrophilicity [34].

**Figure 6:**
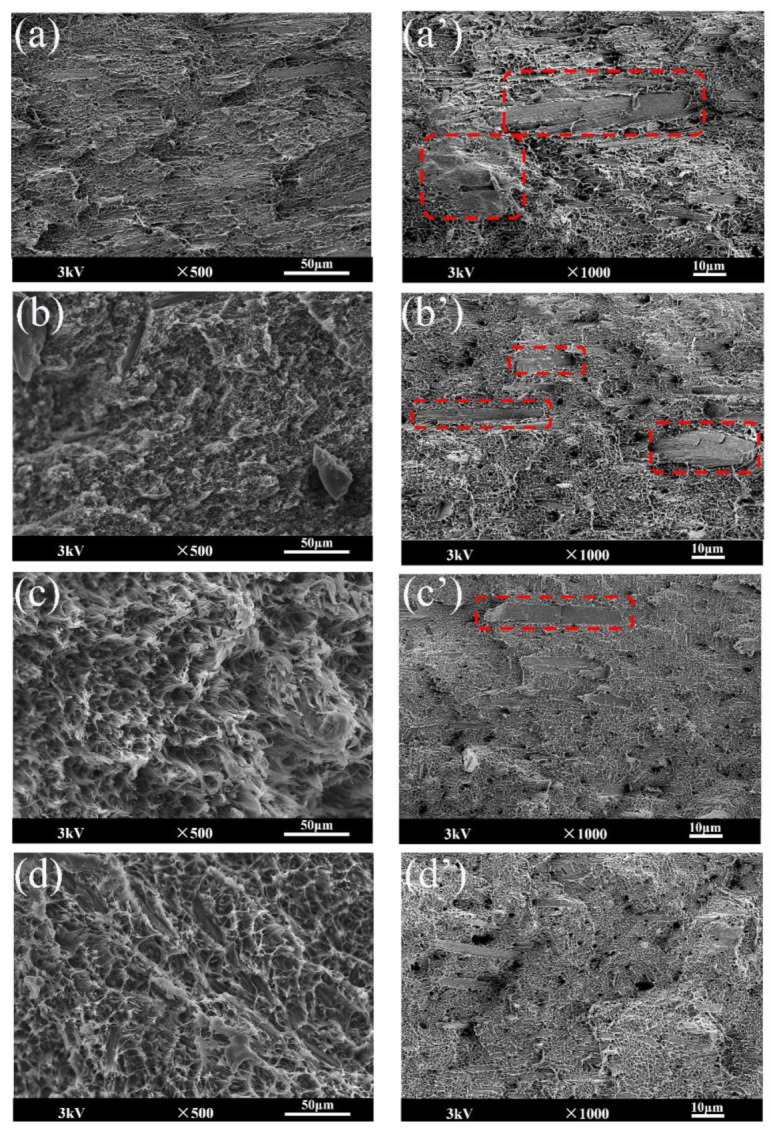
SEM micrographs of (**a**), (**a’**) HDPE/MOSw, (**b**), (**b’**) HDPE/MOSw@SiO_2_, (**c**), (**c’**) HDPE/MOSw@SiO_2_@KH570, (**d**), (**d’**) HDPE/MOSw@SiO_2_@PDA.

SEM images of the composite material are shown in Figure 6. It is accepted that the impact strength of whisker-filled polymer composites was closely associated with several energy dissipation mechanisms, including crack propagation and deflection, breakage of the polyethylene molecular chain, and pulling out of the whisker matrix [35].

As shown in Figure 6a, the section of the unmodified composite mainly showed a brittle fracture state. Due to the poor compatibility between the MOSw and the matrix, the microcrack rapidly propagated around the poor interface areas, yielding the final failure with less energy. Therefore, the impact strength of HDPE/MOSw composites obviously decreased. As shown in Figure 4b, the impact strength of the composite treated with silica was slightly improved. However, as shown in Figure 6b, the impact section of the composite still showed a brittle fracture state. Compared with Figure 6a, the fracture surface shown in Figure 6b is rough and uneven. The possible reason is that the surface treatment of silica slightly improved the compatibility between the whisker and the matrix, which caused the propagation of microcracks to need more energy.

As shown in Figure 4b, the impact strength of the composites after secondary treatment was significantly improved. Figure 6c–d shows that dimples appeared on the fracture surface of the composites due to the whisker pulling-out effect, showing a state of ductile fracture. Due to the strong interfacial bonding force between the modified whisker and the resin, the process of interface destruction consumed a large part of the energy of the microcracks, so that the effect of the microcracks on the whiskers was reduced. At this time, the whiskers were not easy to break under the action of micro-cracks. Instead, after the micro-cracks had destroyed the interface, the whiskers were pulled out from the resin matrix, and the pulling process performed work due to friction, thereby further consuming the crack energy.

The brittle fracture surface morphology of the composite material is shown in Figure 6a‘–d’. The red boxes in the figures show the whisker agglomeration in the matrix. As shown in Figure 6a’, the agglomeration phenomenon of the unmodified whisker in the matrix was serious, which was mainly attributed to the high surface energy of the whisker. The whisker treated with SiO_2_ still had a lot of agglomeration in the matrix. However, compared with the unmodified whisker, the size of the modified whisker agglomeration was relatively reduced. The whisker treated with SiO_2_-KH570 and SiO_2_-PDA showed good dispersion (agglomeration still existed, as shown in Figure 6c’, but the scale was not large). This was mainly attributed to the use of KH570 and PDA, which reduced the surface energy of the whisker and made the whisker less prone to agglomeration. The DSC test results also showed that the dispersion of the modified whisker in the matrix was improved. In addition, since the modified MOSw had better dispersibility in the matrix, it could effectively reduce the occurrence of stress concentration and block the expansion of cracks, thereby delaying the formation of fractures in the composite materials. Therefore, the impact strength of the composites showed an obvious upward trend.

However, compared with the HDPE/MOSw@SiO_2_@PDA composite (Figure 4b and Figure 6c), the HDPE/MOSw@SiO_2_@KH570 composite showed higher impact strength and a more severe matrix deformation state. The fundamental reason was that the introduction of PDA was performed more to enhance the rigidity of the polymer, as shown in Figure 4a; however, the HDPE/MOSw@SiO_2_@PDA composites exhibited the highest Young’s modulus. In addition, DMTA analysis showed that the secondary treatment of PDA significantly improved the compatibility between the whisker and the matrix. However, due to the stronger interfacial binding force, the whisker was difficult to pull out from the matrix, thus reducing the influence of the whisker pull effect on toughness. Therefore, the impact strength of the HDPE/MOSw@SiO_2_@PDA composites was lower than that of the HDPE/MOSw@SiO_2_@PDA composites.

### 3.5. Dynamic Mechanical Thermal Analysis

**Figure 7:**
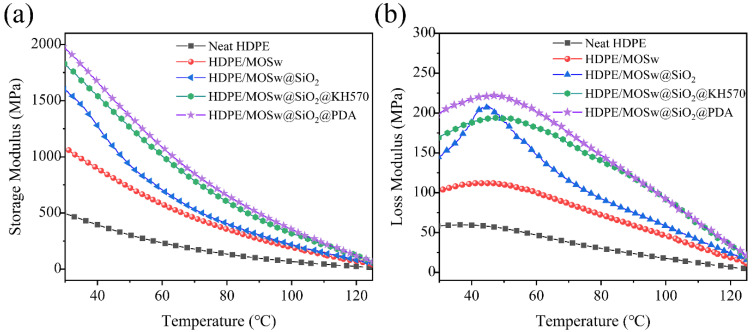
Schematic diagram of dynamic mechanical analysis of the composite materials.

The DMTA curves of HDPE and its composites are presented in Figure 7. The storage modulus(*E’*) is used to describe the elastic part of a viscoelastic material, similarly to the elastic modulus, indicating the material’s ability to resist deformation and store energy. As shown in Figure 7a, the addition of the whisker significantly increased the energy storage modulus of the HDPE composite, which was attributed to the high strength and high modulus of MOSw, and the whisker addition occupying the space between the polyethylene molecular chain, restricting the movement of the molecular chain, and thus hindering the deformation of the composite [36]. The surface coating of silicon dioxide improved the interface bonding force between the whiskers and the resin, so the storage modulus of the HDPE/MOSw@SiO_2_ composite material was increased by 48.6% compared with that of the HDPE/MOSw composite material. The energy storage modulus of the HDPE/MOSw@SiO_2_@KH570 and HDPE/MOSw@SiO_2_@PDA composites was increased by 69.2% and 84.1% compared with that of the HDPE/MOSw composites, respectively. This was mainly attributed to the improvement in the compatibility between the whisker and the resin by the secondary treatment of KH570 and PDA, which further improved the interface binding force between the whisker and the resin so that the stress could be transferred from the resin to the high-performance whisker in time.

The loss modulus (*E″*) refers to the phenomenon of energy loss (transformation) into heat when a material is deformed, reflecting the viscosity of the material. As shown in Figure 7b, the addition of the whisker increased the loss modulus of the composite, which was attributed to the fact that the interaction between the whisker and the resin limits the movement of the polyethylene molecular chain, increasing the viscosity of the composite. The surface coating of silicon dioxide and the secondary treatment of KH570 and PDA further improved the surface roughness of the whisker, making the interface adhesion between the whisker and the resin stronger, and greatly limiting the movement of the PE molecular chain. Therefore, the loss modulus of the HDPE/MOSw@SiO_2_, HDPE/MOSw@SiO_2_@KH570, and HDPE/MOSw@SiO_2_@PDA composites was improved compared with that of the HDPE/MOSw composites. In particular, the sharp relaxation peak of the HDPE/MOSw@SiO_2_ composite appeared at about 50 °C, indicating that the whisker coated with silica had a high degree of restriction on the polyethylene molecular chain in this temperature range.

### 3.6. Differential Scanning Calorimeter Analysis

**Figure 8:**
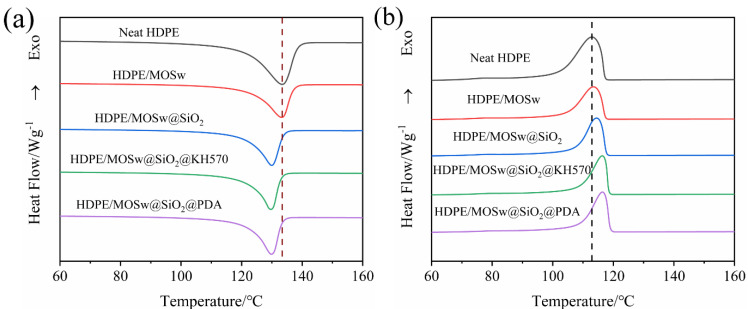
DSC curves of HDPE, HDPE/MOSw, HDPE/MOSw, HDPE/MOSw@SiO_2_, HDPE/MOSw@SiO_2_@KH570, and HDPE/MOSw@SiO_2_@PDA composites.

The DSC curves of the HDPE, HDPE/MOSw, HDPE/MOSw, HDPE/MOSw@SiO_2_, HDPE/MOSw@SiO_2_@KH570, and HDPE/MOSw@SiO_2_@PDA samples are shown in Figure 8. In Table 2, T_c_ is defined as the initial crystallization temperature of the samples, T_p_ is defined as the peak crystallization temperature of the samples, and T_m_ is defined as the melting point of the samples.

The crystallization peak of the composites after filling modification became narrower, and the initial crystallization temperature moved towards a high temperature, indicating that the addition of whisker played an effective nucleation [37] role in the HDPE crystallization process. However, with the introduction of MOSw, the friction between the whiskers and the matrix hindered the movement of the molecular chain, thereby disrupting the highly ordered arrangement of the crystal lattice, so that the crystallinity of the HDPE/MOSw composite material decreased [38]. The surface coating of silicon dioxide increased the surface roughness of the whiskers and further improved the friction between the whiskers and the substrate so that the movement of the molecular chain was reduced, and the crystallinity of the whiskers was significantly reduced. The secondary treatment of KH570 effectively improved the dispersion of whiskers in the matrix, and the well-dispersed whiskers increased the nucleation density of the composite material, which increased the initial crystallization temperature of the composite material from 117.7 °C to 118.5 °C. However, the interface adhesion between the whisker and the matrix after the secondary treatment of KH570 was stronger, which greatly restricted the movement of the molecular chain and further reduced the crystallinity of the composite. The secondary treatment of PDA was similar to KH570, but what was interesting was that the crystallinity of the HDPE/MOSw@SiO_2_@PDA composite was improved compared to HDPE/MOSw@SiO_2_@KH570. It can be inferred that the secondary treatment of PDA was better than that of KH570 in improving the nucleation ability of the whisker in the matrix. This was reflected in the increase in T_c_ for the HDPE/MOSw@SiO_2_@PDA composites compared to that for HDPE/MOSw@SiO_2_@KH570. The higher crystallization temperature enabled the composite material to crystallize at high temperatures, so that the molecular chain could be better stretched to form a more complete crystal, thereby increasing its crystallinity. In addition, the addition of the whisker played a role in heterogeneous nucleation, which improved the nucleation density of the composite material and reduced the crystal size. However, the stability of the small-size crystals was low and they were easily destroyed and formed defects, so the melting point decreased.

### 3.7. TG and Flame Retardant

**Figure 9:**
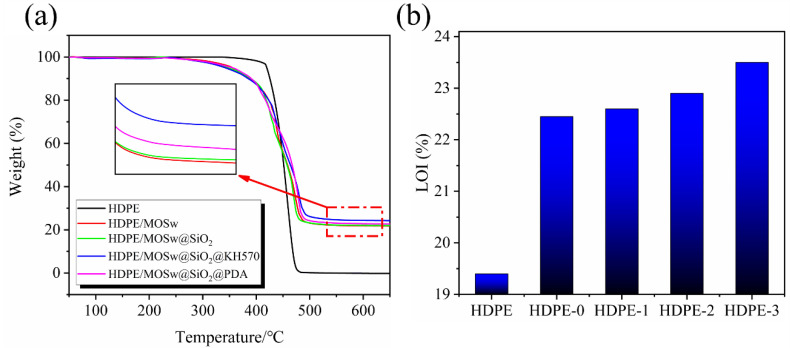
TGA curves (**a**) and LOI data diagram (**b**) of HDPE, HDPE-0 (HDPE/MOSw), HDPE-1 (HDPE/MOSw@SiO_2_), HDPE-2 (HDPE/MOSw@SiO_2_@KH570) and HDPE-3 (HDPE/MOSw@SiO_2_@PDA) composites before and after modification. MgSO_4_·5Mg(OH)_2_·3H_2_O → MgSO_4_·5Mg(OH)_2_ + 3H_2_O (287 °C ~322 °C) (1-1); MgSO_4_·5Mg(OH)_2_ → MgSO_4_ + MgO + 5H_2_O (368 °C ~424 °C) (1-2); MgSO_4_ → MgO + SO_3_ (>800 °C) (1-3).

The TGA curves of the HDPE, HDPE/MOSw, HDPE/MOSw@SiO_2_, HDPE/MOSw@SiO_2_@KH570, and HDPE/MOSw@SiO_2_@PDA samples are shown in Figure 9a. The parameters of thermal stability are summarized in Table 3. Here, T_onset_ is defined as the onset temperature of the sample to decomposition (5% weight loss), and T_max_ is defined as the temperature corresponding to the maximum decomposition rate. Since the dehydration reaction of MOSw began at about 300 °C and the decomposition of polyethylene began at about 420 °C, the initial decomposition temperature of the composites decreased after the addition of MOSw. The T_onset_ of the composites treated by SiO_2_@KH570 decreased significantly, which may have been due to the low degree of the Si-OH polycondensation reaction after KH570 hydrolysis, and the formation of a large number of small molecules on the surface of MOSw. The low thermal stability of the small molecules reduced the initial decomposition temperature of the composites. The significant increase in T_max_ can be interpreted in terms of improved dispersion of the modified whisker in the composite, making the whisker more effective in impeding the thermal movement of the molecular chain, thus improving the thermal stability of the composite [39,40]. Similarly, the T_onset_ and T_max_ of the composites treated by SiO_2_@PDA were improved, which was attributed to the better thermal stability of the PDA itself and the effective dispersion of the modified MOSw in the matrix.

As shown in Table 3, since HDPE was completely decomposed at 650 ℃, the decomposition product of HDPE/MOSw composite was MOSw only, with 21.8% content. The decomposition product of the HDPE/MOSw@SiO_2_ composite was a mixture of SiO_2_ and MOSw, with a content of 22.0%, indicating that the content of SiO_2_ coated on the surface of MOSw was 0.2%. Because the coating treatment of SiO_2_ on the MOSw surface was based on MOSw quality, SiO_2_ with a theoretical content of 1wt.% was prepared (the mass ratio of Na_2_SiO_3_ to SiO_2_ is 2:1). Therefore, it could be determined by calculation that the utilization rate of SiO_2_ reached 20%. As PDA and KH570 perform the role of carbonization at high temperatures and can promote the carbonization of the matrix during the polymer degradation process, their coating quantity could not be effectively calculated by the above method.

As shown in Figure 9b, the LOI of pure HDPE was 19.4. The addition of whisker significantly improved the flame retardant performance of HDPE, and the limiting oxygen index reached 22.5. The reason for the increase in LOI was that MOSw released crystalline water through thermal decomposition while absorbing a large amount of heat through the evaporation of water vapor. The thermal decomposition products covered the surface of the polymer, which effectively isolated the heat and hindered the decomposition of the polymer. Although the endothermic decomposition process of MOSw consumed heat by releasing a large amount of water (Reaction Equation: 1-1, 1-2, 1-3), this discontinuous and loose barrier layer did not effectively prevent the flame from extending into the sample. So, the improved flame retardancy of HDPE/MOSw was dominated by the gas-phase flame-retardant [41] effect of MOSw. The construction of the SiO_2_@KH570 and SiO_2_@PDA double-layer structure formed a stable carbon-silicate layer on the surface of the whisker during the combustion process, which delayed the combustion process. Therefore, the limit oxygen index of HDPE/MOSw@SiO_2_@KH570 and HDPE/MOSw@SiO_2_@PDA composites was improved. The limit oxygen index of the HDPE/MOSw@SiO_2_@PDA composites increased significantly, which was mainly attributed to the scavenging effect of catechol functional groups [42] in PDA, which limited the fuel supply.

## 4. Conclusions

In this paper, MOSw@SiO_2_, MOSw@SiO_2_@KH570, and MOSw@SiO_2_@PDA were successfully prepared from sodium silicate, KH570, and dopamine. Since the Si-OH on the whisker surface was replaced by the hydrophobic group of the silane coupling agent KH570, MOSw@SiO_2_@KH570 showed high hydrophobicity and its contact angle reached 140.7°. However, the secondary treatment of PDA introduced a large number of hydrophilic groups, so the surface of MOSw@SiO_2_@PDA still showed high hydrophilicity. XRD analysis showed that the use of SiO_2_, dopamine, and KH570 did not change the structure of MOSw. DMTA analysis showed that the drag effect of the modified MOSw on the HDPE molecular chain was stronger, which was mainly attributed to better compatibility between the whisker and the matrix. The increase in initial crystallization temperature indicated that the nucleation density of the composites increased, which was beneficial to grain refinement and improvement in the mechanical properties of the composites. Good compatibility between the modified whisker and the matrix was observed in the fracture surface SEM of the composite material, with most of the energy of the microcracks being consumed in the interface failure process. In addition, after the micro-cracks destroyed the interface, the whiskers were pulled out from the resin matrix, and the pulling process also performed work due to friction, thereby further consuming the crack energy. Therefore, the mechanical properties of the composites were obviously improved. The tensile strength of the HDPE/MOSw@SiO_2_@KH570 and HDPE/MOSw@SiO_2_@PDA composites was 22.6% and 41.5% higher than that of the HDPE/MOSw composites, respectively. The impact strength of the HDPE/MOSw@SiO_2_@KH570 and HDPE/MOSw@SiO_2_@PDA composites was 129% and 102% higher than that of the HDPE/MOSw composites, respectively. A stable carbon-silicate layer constructed by the SiO_2_@KH570 and SiO_2_@PDA double-layer structure delayed the combustion process. Therefore, the limiting oxygen index of the HDPE/MOSw@SiO_2_@KH570 and HDPE/MOSw@SiO_2_@PDA composites increased from 22.5 to 22.9 and 23.5, respectively.

## Figures and Tables

**Table 1:** Changes in the elemental content of whisker before and after modification.

	MOSw	MOSw@SiO_2_	MOSw@SiO_2_@KH570	MOSw@SiO_2_@PDA
∆Si	─	+ 4.91%	+ 5.98%	+ 2.89%
∆N	─	─	─	+ 4.03%
∆C	─	─	+ 2.18%	+ 7.66%

**Table 2:** DSC data for HDPE, HDPE/MOSw, HDPE/MOSw, HDPE/MOSw@SiO_2_, HDPE/MOSw@SiO_2_@KH570, and HDPE/MOSw@SiO_2_@PDA.

Samples	T_c_ (°C)	T_p_ (°C)	T_m_ (°C)	∆H (J/g)	Xc (%)
Neat HDPE	117.2	112.9	133.4	200.4	72.3
HDPE/MOSw	117.4	113.4	133.2	135.8	70.1
HDPE/MOSw@SiO_2_	117.7	114.5	130.0	129.3	66.7
HDPE/MOSw@SiO_2_@KH570 HDPE/MOSw@SiO_2_@PDA	118.5 119.0	116.3 116.4	129.7 129.9	126.7 127.3	65.3 65.6

**Table 3:** LOI and parameters of thermal stability for neat HDPE, HDPE/MOSw, HDPE/ MOSw@ SiO_2_, HDPE/MOSw@SiO_2_@KH570 and HDPE/MOSw@SiO_2_@PDA.

Sample	T_onset_ (°C)	T_max_ (°C)	Residue (%)	LOI (%)
HDPE	420	462	-	19.4
HDPE/MOSw	353	436	21.8	22.5
HDPE/MOSw@SiO_2_	341	467	22.0	22.6
HDPE/MOSw@SiO_2_@KH570	329	468	24.3	22.9
HDPE/MOSw@SiO_2_@PDA	355	478	22.7	23.5

## Data Availability

Not applicable.
